# Potential Sustainable Properties of Microencapsulated Endophytic Lactic Acid Bacteria (KCC-42) in* In-Vitro* Simulated Gastrointestinal Juices and Their Fermentation Quality of Radish Kimchi

**DOI:** 10.1155/2018/6015243

**Published:** 2018-09-03

**Authors:** Chae Eun Song, Han Hyo Shim, Palaniselvam Kuppusamy, Young-IL Jeong, Kyung Dong Lee

**Affiliations:** ^1^Lifelong Education Center, Chonnam National University, Kwangju 500-757, Republic of Korea; ^2^Department of Biotechnology, Sunchon National University, Suncheon, Jeonnam 540-742, Republic of Korea; ^3^Grassland and Forage Division, National Institute of Animal Science, Rural Development Administration, Cheonan 330-801, Republic of Korea; ^4^Biomedical Research Institute, Pusan National University Hospital, Busan 49241, Republic of Korea; ^5^Department of Oriental Medicine Materials, Dongsin University, Naju 520-714, Republic of Korea

## Abstract

The objective of this study was to investigate alginate microencapsulated lactic acid bacteria (LAB) fermentation quality of radish kimchi sample and its potential survivability in different acidic and alkaline environments. Initially, we isolated 45 LAB strains. One of them showed fast growth pattern with potential probiotic and antifungal activities against* Aspergillus flavus* with a zone of inhibition calculated with 10, 8, 4mm for the 4th, 5th, and 6^th^ day, respectively. Therefore, this strain (KCC-42) was chosen for microencapsulation with alginate biopolymer. It showed potential survivability in* in-vitro* simulated gastrointestinal fluid and radish kimchi fermentation medium. The survival rate of this free and encapsulated LAB KCC-42 was 6.85 × 10^5^ and 7.48× 10^5^ CFU/ml, respectively; the viability count was significantly higher than nonencapsulated LAB in simulated gastrointestinal juices (acid, bile, and pancreatin) and under radish kimchi fermentation environment. Kimchi sample added with this encapsulated LAB showed increased production of organic acids compared to nonencapsulated LAB sample. Also, the organic acids such as lactic acid, acetic acid, propionic acid, and succinic acid production in fermented kimchi were measured 59mM, 26mM, 14mM, and 0.6mM of g/DW, respectively. The production of metabolites such as lactic acid, acetic acid, and succinic acid and the bacteria population was high in microencapsulated LAB samples compared with free bacteria added kimchi sample. Results of this study indicate that microencapsulated LAB KCC-42 might be a useful strategy to develop products for food and healthcare industries.

## 1. Introduction

Lactic acid bacteria (LAB) are Gram-positive bacteria belonging to order Lactobacillales that produce lactic acid as the major metabolic end product of carbohydrate fermentation. Therefore, LABs have been used for food fermentations because lactic acid can inhibit the growth of spoilage agents. Some LABs have been used as probiotics. Functional and safety profiles of probiotics have been measured by various biochemical parameters such as antibiotic susceptibility, adhesion of intestinal mucosa, digestion and detoxification, production of bioactive metabolites, and the potential survivability of stomach and bile acids [1, 2]. At present, LAB are considered as the normal populace of the GIT (gastrointestinal tract). Therefore, LAB strains are preferably used as probiotics in human and animal foods. Many potential probiotics strains are being used as new frontiers in medicine for human healthcare and livestock industries. FDA has approved different probiotics products. They are available in commodity and commercial markets as foodstuff and ingredients. Most probiotics strains are LAB and bacteria belonging to the genus* Bifidobacterium*. Among various LABs, those belonging to genus* Lactobacillus* represent major uses in food and agricultural industries. Also, LAB strains can produce antimicrobial compounds such as organic acids, fatty acids, peptides, and bacteriocins in fermented food products [3].

Microencapsulation is a packaging technology for solid, liquid, and/or gaseous substance using thin polymeric materials to form small particles called microcapsules. These microcapsules might be broken by different external stimuli such as diffusion, heat, and pressure in the digestive tract, thus releasing active ingredients at the active site in the digestive tract [4, 5]. Different biological materials that are acid liable can be stabilized in gastrointestinal fluids by using coating substances to pass through the acidic condition of the stomach. Therefore, encapsulated bacteria can survive acidic and alkali conditions during intestinal transition [6]. In this work, we isolated a LAB strain from white clover legumes and determined its potential probiotics properties. Also, the isolated strain was microencapsulated with alginate beads, and its survivability in* in-vitro* simulated gastrointestinal fluid was examined. In addition, fermentation quality of radish kimchi added with such microencapsulated LAB strain was determined in this study.

## 2. Experimental Study

### 2.1. Isolation and Identification of Endophytic Lactobacillus Strains

Isolation and identification of LAB strains followed those described previously by Valan Arasu et al. (2014) [7] with minor modifications. Three types of silage samples including hairy vetch, red clover, and white clover were used for the isolation of novel LABs. A 10g of each sample was weighted aseptically condition and 100ml of MRS broth was added. The broth was kept in a shaker 120rpm at 37°C for 24 hrs. After that, the serial dilutions were made for each sample and appropriate volume was evenly spread onto the MRS agar plate. Milky white single colonies were grown and selected (based on color, shape, size, and rough or smooth surface) and then purified using streak plate method by same agar medium. The purified strain was numbered KCC-42 and supplemented with 40% (V/V) glycerol stock and stored at -20°C for further studies. Then, the isolated genomic DNA samples were sent to Macrogen (Seoul, Korea) for 16srRNA sequencing and then obtained sequence was manually aligned and compared with previously published sequences of bacterial strains in NCBI BLAST online database system.

### 2.2. Growth Profiles of Isolated LAB Strains

Growth profiles of isolated LAB strains were studied using MRS broth and IRG (90%) extract. Briefly, 100 *μ*l each isolated LAB culture was poured into MRS broth containing IRG extract and incubated at 37°C in a rotary shaker (1×g) rpm for 24 hrs for growth analysis. After incubation, aliquots (300 *μ*l) of samples were added to 96-well plates in triplicates. The absorbance of the plate was measured at wavelength of 600 nm every 2 hrs for 48 hrs. Values were then used to obtain the growth curve. From a total of 45 colonies isolated, we selected the strain with the fastest growth. The selected strain was named KCC-42 (based on the number the strain was isolated). It was then subjected to evaluation of potential probiotic and functional properties [8].

### 2.3. Antagonistic Properties of Selected LAB

Antifungal activity of the isolated LAB was determined by agar spot method as described previously by Valan Arasu et al. (2014) [7]. Briefly, 10 *μ*l of test culture (KCC-42) and control were spotted onto MRS agar plate followed by incubation at 37°C for 24 hrs. Subsequently, 50 *μ*l of fungal (*A. flavus*,* P. chrysogenum*) spores was collected in 10% triton solution. Each fungal spore was then separately mixed with PDA agar medium and carefully poured over 1 mm layer on bacterial culture spotted MRS agar plate. These plates are then incubated microaerobically at 37°C for 48 hrs. After incubation, antifungal activity was measured by inhibition zone around bacterial spots. Zone of inhibition was measured in millimeters scale.

### 2.4. Antibiotic Susceptibilities of Isolated Strain

Antibiotic susceptibilities of the isolated strain KCC-42 were evaluated by disc diffusion assay using the protocol of the European Food Safety Agency (EFSA). Briefly, MRS agar plates containing 100*μ*l of evenly swabbed bacterial culture of KCC-42 were grown at 37°C for 24 hrs. After that, discs containing standard antibiotics such as ampicillin, gentamicin, kanamycin, streptomycin, erythromycin, clindamycin, tetracycline, and chloramphenicol (Sigma, USA) were placed on MRS agar plates and incubated at room temperature for 10 min to allow diffusion of antibiotics. These plates were then incubated at 37°C for 48 hrs. Inhibition zones were then measured [9].

### 2.5. In-Vitro Hemolytic Activity of Selected Probiotic Strain KCC-42

To determine hemolytic activity of the LAB KCC-42, Colombia agar diffusion method was used. Briefly, a loopful of an overnight culture of LAB strain KCC-42 was taken and streaked onto Colombia agar plates containing 5% fresh blood and incubated at 37 ± 2°C for 36 hrs. Hemolytic activity was observed visually.* Escherichia coli* was used as a control [7].

### 2.6. Autoaggregation Assay for KCC-42

Autoaggregation test was performed according to published method of Reniero et al. (1992) [10] with some modifications. Briefly, the overnight bacterial culture of KCC-42 in MRS broth was centrifuged at 5000 ×g for 10 min. The cell pellet was resuspended in PBS (pH 7) to obtain approximately 10^8^ CFU/ml for auto aggregation assay. After vortexing 3 ml of bacterial suspension for 10s, absorbance was measured at different time intervals (1, 2, 3, and 4 hrs) using a spectrophotometer at wavelength of 600 nm. Self-aggregation ability of bacterial culture was calculated with the following formula: autoaggregation (%) = (1-Abs_t_-Abs_0_) × 100.

### 2.7. Enzymatic Activities of the Selected Strain KCC-42

Enzymatic activities of the isolated LAB (KCC-42) were then evaluated using API ZYM kit (BioMerieux, Inc, USA) according to the manufacturer's instructions. Briefly, 5 ml of freshly cultured bacterial cells was centrifuged at 3000×g for 10 min and the pellet was suspended in sterile dis. H_2_O was used to obtain 10^8^ CFU/ml of viable cells. To characterize enzyme production, 50 *μ*l of the cell suspension was added into each well of the strip followed by incubation at 30°C for 4 hrs. After incubation, 20 *μ*l of ZYM-A and ZYM-B reagent was added to each well followed by incubation at 30°C for 5 min. Positive or negative enzyme activity was then determined [9].

### 2.8. Microencapsulation of Selected LABKCC-42 Using Alginate Beads

Overnight LAB culture was harvested by centrifugation at 3000 ×g for 20 min and washed with PBS. Cells were then resuspended in sodium alginate-saline (1.2% wt/vol). The suspension was then added into a solution of HEPES-buffered calcium chloride 13 mM HEPES, 1.5% (wt/vol) CaCl_2_ (pH 7.4) (Sigma Chemical Co.) containing chitosan 1 mg/ml using a 23 Gauge needle and allowed to form gel for 20 minutes. Chitosan was used to reinforce alginate microcapsule. Alginate beads were washed three times with HEPES solution (13 mM) and lyophilized for two days as described previously [11].

### 2.9. Survival Properties of Selected Strains under Different Conditions

Survival abilities of encapsulated and nonencapsulated LAB were then analyzed under different environments (simulated gastric and intestinal fluid). Previously freeze-dried LAB KCC-42 was subjected to colony counting in MRS agar by pour plate method. Then 0.5 g of microencapsulated LAB strain was dissolved in 4.5 ml of sterile saline and exposed into freshly prepared simulated gastrointestinal fluid (SGF) (pH 1.9) containing pepsin in saline water. Aliquots samples were taken at different time intervals (30, 60, 90, and 120 min) to determine the number of colonies by MRS agar plating method. Bile and pancreatin (1g/l and 0.1g/l, respectively) were then added into the SIF and pH value of the sample was adjusted using 0.5 N NaOH followed by incubation at 37°C for 2 hrs. Subsequently, simulated gastrointestinal fluid (45 ml) was prepared in 0.1% pancreatin and 3.6% bile in saline solution. Then 0.5 g of LAB KCC-42 (freeze-dried and resuspended) was added followed by incubation at 37°C for 4 hrs. Once incubation was started, aliquots of the sample were again collected at different time intervals (120 and 240 min). Serial dilution was then carried out followed by MRS agar plating to count the number of survived bacterial colonies [12].

### 2.10. Fermentation Study: Raw Materials Used for Kimchi Preparation

Radish kimchi is the most common type of kimchi consumed in Korea. For this experiment, we purchased radish materials in the commercial market from Songhwan and washed them with warm water to avoid contamination. Weighed radish (300 g) was then chopped into small square pieces, mixed with 100 g of NaCl and 10 g sugar, and kept at room temperature for 20 min for complete saturation. After that, the mixture was added with 30 ml of fermented shrimp juice, 10 mg powdered pepper, 300 g of dried Korean pepper, and one pear fruit. After mixing well, these freshly prepared radish kimchi samples were used for experimental analysis.

### 2.11. pH of Fermented Radish Kimchi Inoculated with Encapsulated and Nonencapsulated LAB KCC-42

The pH of radish kimchi sample inoculated with encapsulated or nonencapsulated LAB strain KCC-42 was measured in duplicates daily using a digital pH meter (Denver Instruments, USA) throughout the fermentation period. Before using the pH meter, it was calibrated using reference standard buffer pH 7 and pH 10. Fermented radish kimchi sample was added with 2 ml of autoclaved deionized water and stirred well before pH measurement [13].

### 2.12. Bacterial Colony Counting for Fermented Radish Kimchi Samples

Microbial counting was performed for radish kimchi samples added with encapsulated or nonencapsulated LAB KCC-42 during the fermentation process (days 0, 2, 4, 8, 12, and 24). For bacterial counting, 1 g of kimchi sample was diluted in 9 ml of sterile distilled water and homogenized for 30 sec. The homogenate was then serially diluted. Each dilution was then used for microbial counting on MRS agar plate. After incubating plates at 37°C for 24 hrs, the number of milky white colonies of the LAB strain was then calculated manually [14].

### 2.13. Sensory Evaluation of Kimchi Sample

Sensory evaluation of radish kimchi samples was performed after 21 days of fermentation (final product). The evaluation panel consisted of 12 members. Each panel member marked the quality of radish kimchi fermentation added with encapsulated or nonencapsulated product. Scoring was performed based on color, texture, and acidity of kimchi samples (Hedonic score range, 1-9: 1-4, dislike; 5-7, good; 8-9, excellent) [15].

### 2.14. Quantification of Organic Acid Production in Radish Kimchi by RP-HPLC

Quantification of organic acid in fermented radish kimchi samples added with encapsulated or nonencapsulated LAB samples was carried out using HPLC-DAD method. Briefly, fermented liquid kimchi sample (5 *μ*l) was taken at different time intervals to determine the production of organic acids. These samples were mixed with 2 ml of distilled water and kept in a shaker for 10 min (120 rpm, 37°C). After filtering with 0.22 *μ*m membrane filter, the sample was injected to HPLC (Agilent Technologies 1100 Series) equipped with a DAD detector using an HPLC HPX-87C column (5 *μ*m, 4 X 250 mm). Mobile phase was 0.005 M H_2_SO_4_ (pH 2) using an isocratic elution. Flow rate was set at 0.6 ml/min. UV-Vis detection of organic acid range was performed at *λ* = 210 nm [16]. Quantity of organic acid was calculated according to the retention time of peaks based on those of reference standards and unknown regression curve factor.

### 2.15. Statistical Analysis

Experimental data were statistically analyzed using SPSS ver 16.0 (SPSS Inc., Chicago, IL, USA). All experiments were performed in triplicates. Results are presented as mean ± SD. The mean difference of each group was assessed by Student's t-test. Statistical significance was considered at* P *< 0.05.

## 3. Results and Discussion

### 3.1. Isolation and 16s rRNA Identification of KCC-42

Potential probiotic strains were isolated from hairy vetch, red clover, and white clover forage crops and rejuvenated with different growth medium. A total of 45 bacterial strains were isolated from these plant sources. Based on their growth profiles in different selective plant juices and their antagonistic activities against pathogenic fungus, one potential isolate named KCC-42 was selected (Figures S1 and S2). It was then subjected to biochemical and molecular identification. For molecular characterization, 16s rRNA of this isolate was partially sequenced. Its sequence similarities with others were analyzed using NCBI BLAST program. Results of BLAST analysis revealed that it shared high sequence identities (99%) with 16srRNA sequence of* Lactobacillus plantarum*. The 16s rRNA partial nucleotide sequence was deposited in the NCBI database and GenBank accession number for strain KCC-42 is MH507073.

### 3.2. Antagonistic Activity of LAB Strain KCC-42

**Figure 1:**
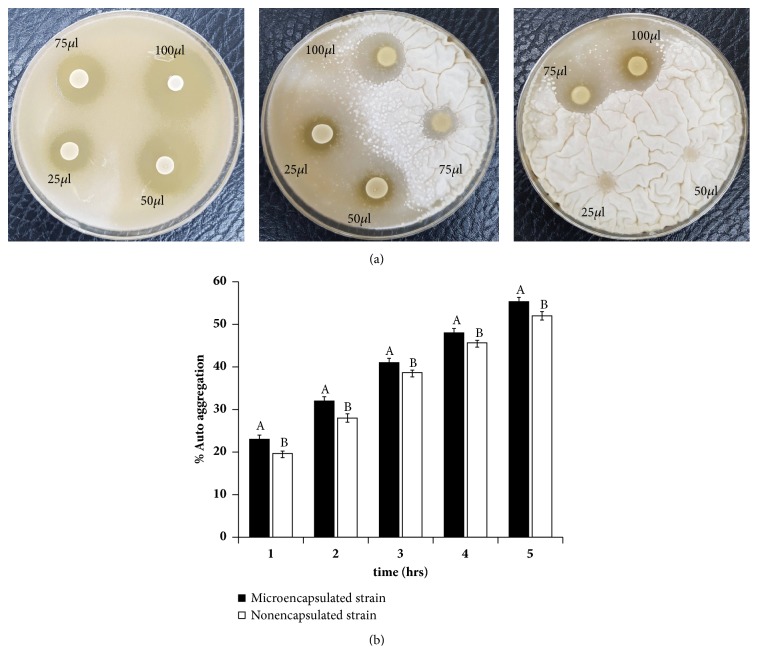
(a) Agar spot test of LAB (25, 50, 75, and 100*μ*l) against pathogenic fungus (*Aspergillus fumigatus*) at days 5, 7 and 9, respectively. (b) Auto aggregation assay of LAB KCC-42.

The antifungal activity of the isolated LAB KCC-42 against different pathogenic fungal strains such as* A. fumigatus*,* A. clavatus*, and* P. chrysogenum* was used. The isolated LAB strain KCC-42 showed various degrees of inhibitory activity against pathogenic fungal spores, and results are shown in Figure 1(a). It is known that LAB can produce organic acid and bioactive metabolites that can inhibit the growth of pathogenic microorganisms. Hence, inhibitory activities of LAB KCC-42 might be directly associated with its ability to secrete organic acid and bioactive compounds in the medium.

Probiotic microorganisms are involved in several health protection mechanisms within the digestive system and internal organs. They are concerned production of pathogen inhibitory metabolites, nutritional competition with pathogenic organisms for the epithelial active site, degradation of toxins and their receptors and modulating the immune response and host, etc. [17]. Also, the oral administration of the microencapsulated probiotics formulated yoghurt could suppress the intestinal inflammation and general functioning which leads to increasing host protection against various pathogens. The supplementation of probiotics is effectively controlled inflammation and for preventing colon cancer. The treated animal groups have provided probiotic microcapsules suspended in saline constantly lower proinflammatory cytokines levels when compared to control group animals [18].

### 3.3. Antibiotic Susceptibility of Selected Strain KCC-42

The* L. plantarum* KCC-42 was sensitive to various antibiotic agents. Results are shown in Table S1. It was found to be very sensitive to most commercial antimicrobial agents such as streptomycin, kanamycin, chloramphenicol, ampicillin, erythromycin, and vancomycin. However, it showed moderate resistance against some antibiotics such as penicillin and erythromycin. Antimicrobial resistance is a challenging issue globally for the healthcare sector, leading to the ineffectiveness of some antibiotics against resistant microorganisms. It has been recognized that antibiotic resistance is due to excessive or inappropriate intake of drugs, leading to less effectiveness and chromosomal resistance of the host. For these reasons, live beneficial bacteria have been used to screen their potential to control harmful pathogenic organisms. Some of them have been found to be more effective in controlling pathogen survival than antibiotics in the host system.* Lactobacillus* strains (POAL: Probiotics Originating from Aloe Leaf) inhibited the growth of many harmful enteropathogens without affecting normal flora in the gut. Moreover, each strain exhibited selective resistance to a wide range of antibiotics. In particular, the overexpression of glutamate decarboxylase (GAD) gene in* L. brevis* could be possible to produce a beneficial neurotransmitter and gamma-aminobutyric acid (GABA) in intestinal system reported by Kim et al. (2014) [19].

### 3.4. Production of Enzymes by the Isolated LAB Strain KCC-42

Microorganisms can produce different kinds of enzymes. Some of them have been used in food products that are beneficial to human health. The LAB KCC-42 could produce important extracellular enzymes such as leucine arylamidase, valine arylamidase, crystine arylamidase, acid phosphatase, naphthol-AS-BI-phosphohydrolase, *β*-galactosidase, *α*-glucosidase, *β*-galactosidase, and N-acetyl- *β*-glucosaminidase (Table S2). Some enzymes such as *β*-glucuronidase are being used for the detection of cancer as a biomarker. However, LAB KCC-42 did not produce *β*-glucuronidase.

### 3.5. Autoaggregation Activity of LAB Strain KCC-42

Cell binding properties of the isolated strain KCC-42 were evaluated by autoaggregation assay. This strain showed potential cell binding properties against food borne pathogens (Figure 1(b)). We also further explored this strain's binding affinities to cell surface and epithelial cells. This* Lactobacillus* strain showed high autoaggregation abilities toward tested pathogens. The adherence ability of LAB has been associated by their surface properties, which consecutively is reflected by the structure, composition, and organization of the cell wall. The cell wall of* Lactobacillus* was made up of a thick peptidoglycan layer, including other surface components such as lipoteichoic acids, polysaccharides, covalently bound proteins, and S-layer proteins. It was previously reported that these components are likely to contribute to the surface properties of a microbial consortium [20].

In the past two decades, bio-organic derived foods are attracting a lot of attention in food and agricultural industries due to their health benefits for human and the environment. Nonpathogenic LABs have been used in the fermentation process naturally. They are strongly resistant to artificial gastric, bile, and intestinal juices. In this study, we evaluated the ability of the novel* L. plantarum *KCC-42 to survive different acid and salt conditions, especially for fermentation of native Korean radish kimchi. Our results revealed that the microencapsulated LAB strain KCC-42 was more stable (having higher number of colonies) than nonencapsulated strain KCC-42. In addition, this* Lactobacillus *strain KCC-42 was able to inhibit the growth of selected pathogens under* in-vitro* condition. It is known that plant-derived LABs have stronger resistance to low pH than animal-derived LAB because their outer membranes are thicker and more solid than their animal-derived counterparts. Therefore, they can adapt to harsh environment conditions more easily. Gao et al. (2012) [21] have reported that one of the important criteria of a probiotic strain is that it has antagonistic activity against harmful pathogens. Probiotics strains might produce some inhibitory substances such as organic acid, bacteriocin, and peptides. These substances have strong interactions with cell walls of pathogenic organisms, thus controlling their transcriptomic function.

### 3.6. Survival Ability of Encapsulated and Nonencapsulated LAB in Simulated Gastrointestinal Conditions

**Figure 2:**
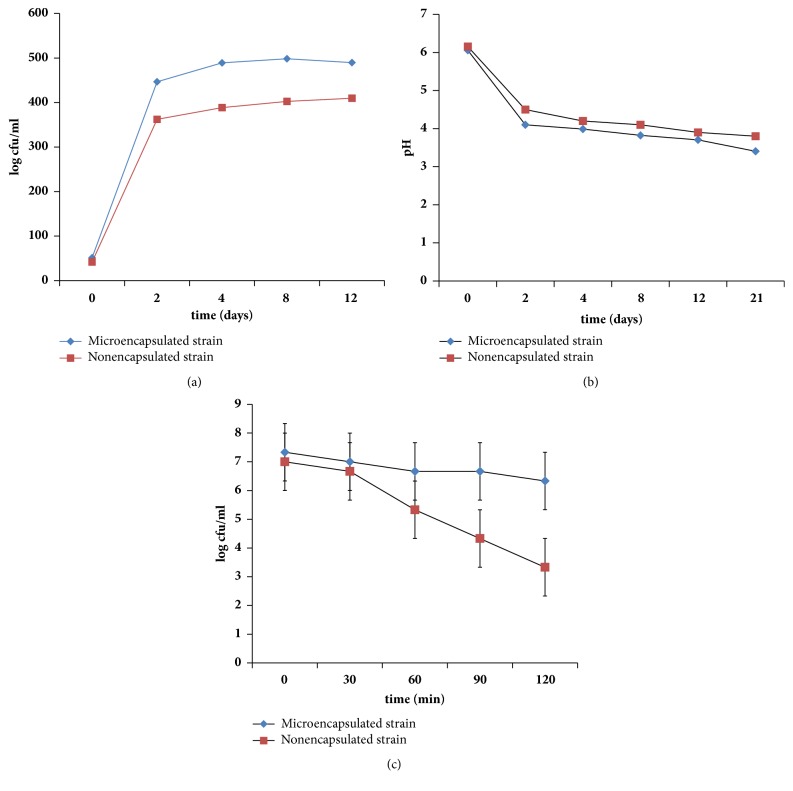
(a) Survival of encapsulated and nonencapsulated LAB during incubation at 37°C in simulated gastric juice (pH1.9) followed by small intestinal juice (pH5-7). (b) Variation pH in radish kimchi samples inoculated with encapsulated and nonencapsulated LAB strain at different time period (days 1, 2, 4, 8, 12, and 24). (c) Bacterial population counting in encapsulated and nonencapsulated LAB inoculated radish kimchi samples during different fermentation period.

After exposing microencapsulated and nonencapsulated LAB KCC-42 to simulated gastric juice at pH 1.9 for 30 min, survival abilities of these LAB were then determined. Encapsulated bacteria were found to have higher survival rate than nonencapsulated KCC-42 after exposure to simulated gastric juice (SGJ) (pH 1.9) for 30 min (Figure 2(a)). The number of survived nonencapsulated bacteria was gradually decreased after 30 min of SGJ exposure. After SGJ treatment, both cells were directly transferred to SIJ (pH 6.5) and exposed for 60 min. Encapsulated cells showed higher viability rate 7.48× 10^5^ CFU/ml and tolerance to high acidic/pancreatin medium compared to nonencapsulated cells 6.50× 10^5^ CFU/ml. This indicated that encapsulated cells could survive the stomach and remain active in the intestine. However, nonencapsulated cells were less resistant to simulated gastrointestinal fluid exposure compared to encapsulated cells of KCC-42. Hence, encapsulated probiotic strain KCC-42 might be useful in kimchi fermentation due to its tolerance to acidic and alkali medium. LAB growth was increased in the acidic environment of* koozh* (pH 4.5) which is made of finger millet and rice that consider as a good substrate for a probiotic carrier molecule. The food matrix enhances survival rate and stability of the LAB against gastric acid damage [22].

LABs have susceptibility against harsh and extreme alkaline conditions in human digestive system. Thus, microencapsulation has been a promising technique for a good and reliable protection of the bacteria in different conditions. Microencapsulation can be protected against cell injury or loss by retaining cells within the encapsulating membrane. Many ways are used to enforce probiotics such as spray-drying, emulsion, extrusion, and other technologies in combination with a suitable coating material such as alginate, chitosan, and combination of other biopolymers [23].

### 3.7. Radish Kimchi Fermentation Properties: pH of LAB Inoculated Kimchi Samples

Results of pH change during kimchi fermentation after adding encapsulated or nonencapsulated LAB KCC-42 at different time intervals are shown in Figure 2(b). The pH of radish kimchi sample added with encapsulated* L. plantarum* KCC-42 was lower than that of kimchi sample inoculated with nonencapsulated LAB KCC-42. The pH of kimchi sample inoculated with microencapsulated LAB strain KCC-42 was decreased to be less than pH 3.4, indicating that this strain could survive an acid environment.

### 3.8. Bacterial Counts in Fermented Kimchi Samples

Encapsulated LAB KCC-42 cells showed good viability in kimchi sample even at pH 3.8. These encapsulated cells showed significantly higher survival rate in fermented radish kimchi sample over three weeks of fermentation period at room temperature (27°C) compared to nonencapsulated cells. The relationship between pH values and viable colonies is shown in Figure 2(c). Microencapsulated cells were more resistant to low pH than nonencapsulated counterparts, with significantly higher number of colonies in LAB encapsulated kimchi sample being noticed (Figure 2(c)). A dry fermented sausage (sucuk) production with free and microencapsulated of* Lactobacillus rhamnosus* was investigated the effect on probiotic viability and quality characteristics on the final product. The sucuk production was developed with the use of microencapsulated and free LAB. The addition of probiotics to the sucuk could contribute to the health benefits and increase in the consumption of such products [24]. The encapsulated* L. acidophilus*,* L. bulgaricus*,* L. lactis*, and* B. bifidum* in orange juice were resistant to decline their viability amount as quickly as the unencapsulated probiotic bacteria and >10^6^ CFU/mL still appeared after 45 days of storage. The free form of probiotics was noticed significant reduction in cell viability in orange juice at both 37°C and 4°C temperatures. Furthermore, the LAB strains were suppressed by the growth of pathogenesis in fermented product due to the production of organic acid such as lactic acid, acetic acid, H_2_O_2_, or bacteriocin-like metabolites [25].

LAB starter culture was mainly stable in the different storage condition at 4-20°C. The* S. thermophilus* and* B. longum* showed a survival percentage of 51.1% and 68.8%, respectively, in dried fermented soymilk stored at 4°C. Under this storage condition, the LAB strains were potent viability in the freeze-dried fermented soymilk after 4 months of storage [26]. The immobilization of* B. infantis* ATCC15697 in alginate or low-methoxylated pectin hydrogel particles significantly increased the survival rate of these strains in fermented nonmilk beverages during storage compared with nonencapsulated cells. Also, the supplementing the microencapsulated bacteria of fermented beverages did not affect the overall sensory quality of beverages during the storage at different temperature conditions [27].

### 3.9. Sensory Evaluation of LAB Inoculated Kimchi Samples

Results of sensory evaluation for kimchi samples inoculated with encapsulated or nonencapsulated LAB KCC-42 cells are summarized in Table 1. There was no significant variance in appearance or color of these kimchi samples. Although kimchi sample inoculated with alginate capsules might show slight changes in color and texture of kimchi, panelist members could not identify any difference in appearance or color of kimchi samples between those inoculated with encapsulated cells and those added with nonencapsulated LAB cells. Corona‐Hernandez et al. (2013) [17] reviewed that the probiotic supplementation to dairy and nondairy products may enhance their sensory attributes. The addition to cheese product with proper culture composition showed no significance changes in the flavor and other sensory characteristic compared to control. However, the probiotic starter culture on cheese could enhance the metabolic activity during production and storage of cheese product. LABs are beneficial bacteria present in most of the fermented food products and produce organic acid as an end product of fermentation process [28].

### 3.10. Quantification of Organic Acid Produced in Fermented Radish Kimchi Samples

Encapsulated LAB KCC-42 tolerated the acidic nature of fermented kimchi samples. Reduction in pH was noticed in both kimchi samples inoculated with encapsulated or nonencapsulated LAB cells, eventually producing organic acid in fermented kimchi samples. Results of quantification of organic acid produced in kimchi samples inoculated with encapsulated or nonencapsulated LAB cells are shown in Table 2 and Figure S3 (A-E). The lactic acid was high amount. 55.6mM g/DW was produced in encapsulated LAN inoculated radish kimchi sample. It was 3-fold higher than nonencapsulated cells added kimchi. Kimchi is a traditional Korean cuisine mainly made of fermented vegetables with a mixture of salt and other side dishes such as pepper, chilli powder, ginger, garlic, and fruits to enhance its flavor and improve the fermentation process which usually takes a few weeks. LAB could naturally enhance functional properties, flavor, and safety of fermented vegetable products. Kailasapathy et al. (2006) [29] have studied yoghurt added with free or encapsulated LAB strains and found that encapsulated strains show lower pH survivability than free strains during the fermentation period. Similarly, Aymerich et al. (2006) [30] have reported that LAB strain is resistant to acid produced condition during fermentation of sausage. After 7 or 14 days of ripening period, the pH was steadily decreased from 5.89 to 5.42 or from 6.16 to 5.50, respectively. Similarly, Cha et al. (2008) [31] have reported natural probiotic flora typically processes kimchi fermentation. They found that LABs isolated from Dongchimi (radish kimchi) have potent tolerance to aciduric and alkali conditions.

Also, kimchi is a famous Korean tradition fermented food using vegetables, it contains various native LAB strains, including* Bacillus sp*.,* Lactococcus *sp.*, Lactobacillus *sp.,* and Weissella *sp. The LAB strains that are involved during kimchi fermentation and different nutritional substance are believed to have possible health benefits for human. Among the several species, the* Lb. plantarum* are present in fermented vegetables due to their potent resistance to survive in high saline and acidity content of fermented vegetables, such as cabbage, cucumber, sauerkraut, and olive [32]. Naturally occurred probiotic organisms in kimchi samples have been found to possess potent anticancer and antioxidant activities. The viability of strain KCC-42 in kimchi sample added with nonencapsulated cells was strongly affected by the reduction in pH. It has been reported that wild microbes such as* Lactobacillus* and* Leuconostoc* bacterial genera play key roles in kimchi fermentation [1]. The LAB utilizing the sugar in anaerobic conditions resulted in producing acids and reducing pH in the fermented samples. The fermented products contain different primary and secondary metabolites which are effectively identified by various analytical techniques such as high performance liquid chromatography (HPLC), LC-MS, and GC-MS. GC-MS is commonly used for metabolic analysis of fermented food samples. Nevertheless, the HPLC has an inferior chromatographic resolution. But it is more convenient technique and measures a wider range of analytes with higher sensitivity [33]. In contrast, the number of total LAB or* L. rhamnosus* in fermentative sample did not show significant differences between control and treated groups after 49 days of the manufacturing process (ripening and storage). Based on RAPD-PCR analysis, bacterial counts of* L. rhamnosus* were found to be increased from 7 log CFU/g to 8.8 log CFU/g during the first seven days of ripening. This level was maintained until the end of the ripening time (day 14, 8.3 log CFU/g) [34]. In addition, the alginate microencapsulated and free form* Lactobacillus paracasei* ssp. was added to part-skim Mozzarella cheese product. The storage at refrigerated condition showed no changes in the free and encapsulated bacterial samples; however, both kinds of cheese bacterial count were decreased significantly during storage period. So, the encapsulation did not increase the survival rate in SGJ containing HCl. On the other hand, it was unexpected that alginate encapsulated cheese product increased the survival rate of* L. paracasei* in H_3_PO_4_ SGJ [35].

Quick drop in pH of kimchi samples might inhibit the growth of spoilage bacteria. We found that the amount of lactic acid produced in kimchi sample inoculated with encapsulated LAB strain KCC-42 pH (pH 3.65) was lower than that in kimchi sample added with nonencapsulated bacteria (pH 4.62). In addition, amounts of acetic acid and succinic acid were slightly different between kimchi samples inoculated with encapsulated bacterial and those inoculated with nonencapsulated bacteria. Fermentation of sugars to provide organic acids such as lactic acid, acetic acid, and butyric acid has been found to be associated with the pH decrease of fermentation medium. Chookietwattana et al. (2014) [36] have reported that organic acid production amount is high when sample pH is more than 6. However, acid production is slow when sample pH is reduced to 5 due to the nature of LAB growth under acidic condition and slowly decreased number of viable counts.

## 4. Conclusions

A wide range of beneficial bacteria are present in plant sources, particularly forage crops. They might have potent applications for food, agricultural, and livestock industries. Among 45 isolated bacteria, we selected a potent LAB strain KCC-42 based on its probiotic properties such as fast growth pattern, antagonistic performance, and antibiotic susceptibility. Microencapsulated LAB strain KCC-42 was found to be able to significantly improve kimchi fermentation quality more than nonencapsulated bacteria cells. Kimchi sample inoculated with encapsulated LAB strain KCC-42 also showed higher increase in the production of organic acids with increasing fermentation time compared to kimchi sample inoculated with nonencapsulated cells. In addition, fermented radish kimchi sample contained other major metabolized products such as lactic acid, acetic acid, butyric acid, and succinic acid secreted by this LAB strain. Therefore, microencapsulated LAB could be useful for food and beverage industries as an alternative of chemical preservative during food processing process.

## Figures and Tables

**Table 1:** Sensory test of LAB KCC-42 inoculated radish kimchi samples.

**Samples**	**Fermented radish kimchi**		
	**Colour**	**Smell**	**Taste**
Encapsulated LAB	5.45	7.65	7.85
Nonencapsulated LAB	5.10	6.74	7.15

**Table 2:** Organic acid production from encapsulated and nonencapsulated LAB inoculated radish kimchi samples after 21 days of fermentation at 37°C.

**S.No**	**LAB strain KCC-42**	**Lactic acid(mM)**	**Acetic acid(mM)**	**Succinic acid(mM)**	**Propionic acid(mM)**	**Oxalic acid(mM)**
1	Microencapsulated LAB	59	26	14	0.6	0.3
2	Non encapsulated LAB	42	21	0.8	0.3	0.2

## Data Availability

The data used to support the findings of this study are available from the corresponding author upon request.

## Supplementary Materials

Supplementary Materials Table S1 shows the antibiotic susceptibility of isolated LAB KCC-42. Table S2 indicates that extracellular enzymes production by LAB KCC-42 is identified by using the API ZYM kit method. Figure S1 reveals the growth pattern of isolated LAB strains in IRG plant juice. Figure S2 shows comparison of antagonistic test of isolated LAB strains (total 45 strains) against fungus A. fumigates. Figure S3 shows the HPLC Chromatograms of organic acid production in encapsulated and nonencapsulated LAB inoculated cabbage kimchi samples after 2 and 21 days of fermentation time at 37°C.
